# Three Cases of Paracentesis Thoracis in Pleurisy; Two of Which Were Successful

**Published:** 1852-09

**Authors:** Joseph Beyran


*Three Cases of Paracentesis thoracis in Pleurisy; two of which were successful.* By Dr. Joseph Beyran.—Dr. Beyran is in favor of puncturing the thorax at a comparatively early period in pleurisy. He sometimes employs a trocar, sometimes a bistoury; the latter plan he prefers when the subject is fat or muscular. The three cases to be related occurred in Constantinople; two of them in a hospital there, to which Dr. Beyran is attached.

CASE 1. Margos, aged 28, a sawyer, exposed to alternations of heat and cold, was seized with pleurisy on February 3d, 1851. On the 9th he was admitted into the hospital, with signs of effusion in the right pleural sac. On the 11th, the respiration becoming more and more impeded, Dr. Beyran punctured the thorax between the seventh and and eighth ribs, by means of the trocar of M. Reybard. Six and a half pints of transparent serous fluid escaped. A piece of diachylon plaster and a bandage were applied. After the operation he gradually recovered, and was discharged cured on February 26th; and has since remained well.

CASE 2. Serpoulic, aged 32, a washerwoman, had been ailing for some days, with a pain in the left side of the chest, when she accidentally burnt her legs on April 13th, and was brought to the hospital. The next day, the most urgent symptoms were those of extensive effusion into the left pleura. Blisters and diuretics were used, without effect. On the 16th, as the patient was threatened with suffocation, Dr. Beyran punctured the thorax. The patient being fat, an incision was made first down to the ribs, until fluctuation was distinctly felt with the finger; the bistoury was then introduced, and five pints of yellow transparent fluid were removed. The flow was then suddenly arrested to prevent the access of air; plasters, graduated compresses and a bandage were applied. In half an hour the stroke-sound was normal, except at the lower part; and the respiratory murmur could be heard. The patient was calm. The next day she was much better; and on August 18th she left the hospital completely recovered.

CASE 3. Dr. Beyran was called in consultation in the case of Mlle. M. Narisen, who was seized on December 2d, with pleurisy of the right side. He saw her on the 12th, when he diagnosed effusion into the right pleural sac. The patient also had tenderness in the hypochondria and epigastrium, with nausea and vomiting. Dr. Beyran recommended paracentesis; but could not prevail on the other medical attendants to have it performed until several days after. It was then performed, when suffocation was imminent, by puncture with a bistoury. The patient appeared relieved for a time, but died exhausted in eight hours after the operation. Before her death, she complained of much pain in the region of the stomach.—*Lond. Journ. Med.*

